# Phenomenology of the COVID-19 Pandemic Experience in Patients Suffering from Chronic Schizophrenia—A Qualitative Analysis

**DOI:** 10.3390/ijerph19010056

**Published:** 2021-12-22

**Authors:** Katarzyna Kotlarska, Benita Wielgus, Łukasz Cichocki

**Affiliations:** 1 Institute of Psychology, Pedagogical University of Cracow, ul. Podchorążych 2, 30-084 Kraków, Poland; 2 The Education of Research and Development Center, Babinski Clinical Hospital, ul. Babińskiego 29, 30-393 Kraków, Poland; benita.wielgus@gmail.com (B.W.); lwcichocki@gmail.com (Ł.C.); 3 Department of Psychiatry, Andrzej Frycz Modrzewski Krakow University, ul. Herlinga-Grudzińskiego 1, 30-705 Kraków, Poland

**Keywords:** schizophrenia, COVID-19, pandemic, experience, isolation, qualitative analysis

## Abstract

Many studies have shown that the COVID-19 pandemic can have a great influence on mental health. However, there is still not enough research to fully understand how people suffering from schizophrenia experience crisis situations such as a pandemic. This qualitative study aims to explore this subject. Ten outpatients suffering from schizophrenia were interviewed in a semi-structured format using an interview designed by the authors for the purpose of this study. The interviews were transcribed, and a conventional qualitative content analysis was conducted. The general themes identified in the content analysis were organized into four categories: first reactions to information about the pandemic; subjective assessment of the pandemic’s impact on patients’ mental health; patients’ attitudes towards the temporary limitations and lockdowns; psychiatric treatment and psychotherapy during the pandemic. A variety of different experiences were observed, but the general conclusion arising from the study suggests that the majority of the interviewed patients coped quite well with the pandemic and that the observed reactions were similar to the reactions of other groups described in the literature. The study also confirmed the importance of the continuity of psychiatric care for patients with schizophrenia.

## 1. Introduction

Since the World Health Organization (WHO) announced the COVID-19 pandemic, the whole world has struggled not only with the threat to people’s health and lives but also with many negative social and economic consequences [1]. In Poland, the first restrictions, the so-called “lockdown”, were introduced in March 2020 and included, similarly to other countries, movement bans in public areas, social distancing, and the necessity of wearing face masks [2]. Infected and potentially infected people were quarantined. The applied security measures can reduce SARS-CoV-2 transmission, but it is important to remember that they can also affect emotional states, causing feelings of uncertainty, anxiety, and disorientation [3]. Some highly vulnerable groups, including the elderly, women, minorities, and people with medical conditions [4] or severe mental illnesses, such as schizophrenia [5], are more exposed to the influence of these limitations. Studies show [6,7] that mental health issues in the general population are increasing, including addictions and domestic violence. People with preexisting mental health disorders are a more vulnerable group and may be at risk of developing suicidal ideation or posttraumatic stress disorder [8].

The question that inspired this article was how people suffering from chronic schizophrenia experience the COVID-19 pandemic and the limitations it entails. So far, there has not been enough empirical research concerning the functioning of schizophrenia patients during the COVID-19 pandemic [9]. It has been demonstrated that stress related to the pandemic worsens mental health in the general population [9,10]; therefore, it seems reasonable to expect that the impact might be even greater for people living with schizophrenia [11]. However, most studies focus on the general population (e.g., [12]) or other mental disorders (e.g., [13,14]), so this impact does not necessarily have to be the same for people with schizophrenia. The everyday life of most people has changed due to the pandemic; however, schizophrenia patients had often experienced social isolation on a daily basis before the pandemic [15,16], so they may be more able to comply with social distancing directives and better tolerate the emotional load they cause [11]. On the other hand, it also needs to be considered that there is strong evidence in previous research [16,17,18] of an association between social isolation and worse functioning of people with mental health history.

In general, there are two notions in the literature regarding the influence of stressful events on schizophrenia patients [19]. Many authors (e.g., [20]) emphasize reduced coping abilities due to the psychotic condition, which additionally causes patients to interpret neutral situations as a threat. Early research concerning the COVID-19 pandemic [21,22] assumed that suffering from a mental disorder can be a psychosocial vulnerability when it comes to dealing with the pandemic. On the other hand, some research has shown (e.g., [23]) that during disasters such as fires or war, patients with schizophrenia are focused on survival and do not tend to panic. Research conducted in Israel during the SARS epidemic in 2002–2004 [19] showed that some patients with schizophrenia experienced more anxiety than the control group, and some perceived the epidemic in a psychotic manner. However, many patients’ responses did not differ from the control group. The authors summarize that “it seems that patients attempt to reduce the effect of external stressors by living in an ‘autistic bubble’ or by denying the significance of these stressors” [19] (p. 258).

The risks of COVID-19 are another important issue that should be considered in relation to how schizophrenia patients experience the pandemic. The latest research [24] has shown that people suffering from schizophrenia are at higher risk of COVID-19 infection and experience worse outcomes of this disease. There is also an increased mortality rate from COVID-19 in people with severe mental disorders compared to the general population [25]. Fonseca et al. [24] indicate three aspects of this situation: firstly, due to psychotic symptoms, schizophrenia patients may have more difficulties related to pandemic procedures and restrictions and are thus at higher risk of contamination; secondly, schizophrenia is possibly associated with immunodeficiency, which may cause worse COVID-19 outcomes in schizophrenia patients; thirdly, pandemic restrictions can cause limited access to health care and social support, which means more emotional distress and higher risk of psychotic relapse.

The directives and limitations resulting from the pandemic created challenges for mental health professionals and community services that support patients with schizophrenia [11]. Still, continuity of care is critical for these patients to prevent decompensation and its consequences [26]. In Poland, multiple health care facilities were closed due to the pandemic for at least a few months. Interactions with patients were limited only to phone calls or video conferences, which cannot fully replace the routine of everyday participation in therapeutic classes. This disruption in health care can place patients at risk. For example, social isolation among schizophrenia patients may elevate the risk of suicide [27], and stress may increase aggressive behavior [28].

To sum up, many factors can influence the reactions of schizophrenia patients to the pandemic. The results of this study could provide some preliminary insight into these patients’ personal resources and contribute to better understanding of how they experience the pandemic. A closer look into the subject is greatly needed as there is still a deficiency of research on the quality-of-life determinants of people suffering from schizophrenia [29]. The presented pilot study used qualitative methods to explore the subject.

## 2. Materials and Methods

### 2.1. Participants and Procedure

The study received ethical approval from the Research Ethics Committee of the Pedagogical University of Krakow. Ten participants were recruited by professional workers in outpatient services (Mental Health Clinic and Day Ward Unit) at the Babinski Hospital in Krakow, Poland to take part in interviews about their experiences during the pandemic. The inclusion criteria were as follows: (1) age above 18 years, (2) diagnosis of schizophrenia with minimum two-year course of the disease, (3) lack of acute psychotic symptoms, (4) participation in outpatient therapy. All patients were under a psychiatrist’s care, who assessed the patient’s capability to take part in the study. Participants were informed about the study procedure and were asked to give informed consent to participation before the interview. In the Day Ward Unit, before the interview, patients signed the consent to take part in the study, consent to record an interview, and the General Data Protection Regulation. In the Mental Health Clinic, interviews were performed via phone calls due to security measures introduced in the Hospital in regard to the pandemic situation. In this case, consent was audio recorded before starting the conversation. Additionally, participants were asked to sign all the above documents during their next doctor’s appointment. Interviews were audio recorded separately from the participation consent and transcribed in compliance with data protection regulations. Participants could withdraw their consent at any time during the interview and research. The interviews lasted 20 to 40 min. Data were collected from September 2020 until January 2021. During this time, in Poland there was a rapid increase in new COVID-19 cases from 700 per day in August 2020 to 27,000 per day in November 2020. The mortality rate was approximately 2.4%. A “red zone” was introduced in all of Poland, so the restrictions were strict. Most public facilities were closed or worked online. For some time, the situation in public health care was critical. However, some of the mental health outpatient services were open. The vaccinations had not started yet.

### 2.2. Interviews

The interview used in the study was semistructured and was constructed by the authors. The selection of the questions was discussed among the authors and two independent consultants in relation to the aspects of experience described in detail in Table 1 The interview covered several topics concerning general information (first part) and the pandemic experience (second part). The first part of the interview included demographic questions and focused on principal information about the course of illness and treatment, mental health, and general wellbeing before the COVID-19 pandemic. The second part related to how the patients experienced the beginning of the pandemic and what feelings or thoughts were associated with it. The questions primarily covered changes in their lives as a result of pandemic limitations, including subjective impact on mental health and interpersonal functioning, as well as psychiatric or psychological treatment during the pandemic and patients’ opinions and feelings about professional support and treatment possibilities during the pandemic. The translation of the interview questions concerning the pandemic experience are presented in Table 1. The interview was semistructured, so in some cases, the questions were asked in different order or some follow-up questions were added to keep the conversation with the participant more fluent and natural.

### 2.3. Analysis

All audio-recordings from the interviews were transcribed into Microsoft Word. A conventional qualitative study [30] was conducted based on the interpretative phenomenological analysis [31]. Transcriptions were read carefully several times in order to thoroughly understand the participants’ meanings. The next step was coding [32], which involved seeking significant phrases that indicated how patients experienced the pandemic. Two-level coding was applied. The first level involved marking particular patients’ responses with codes associated with the main content of the response. The expected aspects of patient’s experience described in Table 2 were taken into consideration. The second level included grouping the responses into categories. It was possible to extract some important topics which all participants brought up at some point during the interview. These identified themes were organized into four categories:

1. First reaction to information about the pandemic.
2. Subjective assessment of the pandemic’s impact on patients’ mental health.
3. Patients’ attitudes towards temporary limitations and lockdowns.
4. Psychiatric treatment and psychotherapy during the pandemic.

Citations from the discourses were translated from Polish into English and added to the analysis as examples of specific categories.

## 3. Results

The mean age of participants was 36.70 (SD = 4.989); 30% of the sample was women (mean age of women was 39 and mean age of men was 35.7). None of the participants were hospitalized during the pandemic. Table 2 shows the sociodemographic characteristics of the sample.

In the interviews, patients referred to four main topics which are described in the Methods section and seem relevant to how patients experience the pandemic situation. All the above topics were analyzed in reference to the patients’ mental health history, functioning before the pandemic, and perceived level of social support.

### 3.1. First Reaction to Information about the Pandemic

In general, there were three kinds of initial reactions to the pandemic: anxiety (P2, P3, P6, P8), depression (P5), and indifference/disbelief (P1, P4, P7, P9, P10).

Four patients reported feeling anxiety and uncertainty associated with the possibility of contamination (themselves or others). At first, they thought the risk of contamination or death was much higher than it really was at that time. Two of these patients had a stable job; one was a regular participant of occupational therapy workshops.

P2: “*I bought a lot of disinfectants. I disinfected the door handles because I was afraid. Most of all, I was afraid that I might infect my mother and that even if I didn’t have symptoms, I might be spreading these germs*”.

P3: “*In the beginning I caught a cold and just panicked and ran to the Emergency Room because I thought I had the coronavirus”.*

P6: “*I was afraid of being infected. I am at increased risk because of my diabetes. It was hard to leave the house; I thought that I might die”.*

One patient reported depressive symptoms related to the pandemic restrictions. This patient used to be very active before the pandemic, had a job, and had not experienced severe psychotic symptoms for many years. 

P5: “*Well, I was so broken and depressed at first because I liked traveling so much and it felt like these limitations just broke me a bit because I had to stay at home. I picked myself up quickly and later I felt ok”.*

Five patients said that at first they were indifferent and just accepted the situation or could not believe that the pandemic might affect them in any way. All of these patients did not have a job before the pandemic. 

P4: “*I didn’t care too much because I had a more stay-at-home lifestyle anyway*”.

P7: “*At first I did not believe that there was such a thing. I was half serious and half joking about it*”.

### 3.2. Subjective Assessment of the Pandemic’s Impact on Patients’ Mental Health

Most of the patients reported that the pandemic had some negative impact on their mental health, though only two of them (P6, P10) had a psychotic relapse during the pandemic.

P6: “*It got worse because I had to stay at home, I couldn’t find a place for myself; I was nervous. I didn’t leave the house at all, so I started having delusions*”.

P10: “*Things started to get a little weirder. I’m just oversensitive to things that seem strange to me in the world. When something weird happens, like this pandemic, I just start taking it so much more personally and I start to have some irrational thoughts*”.

Other patients noticed anxiety or depressive symptoms. P6 had a relapse after the occupational therapy workshops had been closed due to the pandemic. P2 started working online during the pandemic and noticed the negative impact of isolation on her mood and personal hygiene. P8 noticed that the lockdown had an impact on his typical coping strategies (going to the gym, travelling), which caused feelings of isolation and insecurity. One patient (P1) remained indifferent to the pandemic and another patient (P5)—after experiencing initial depressive symptoms, became indifferent after getting used to the pandemic limitations.

It also seems significant that six patients (P3, P4, P6, P8, P9, P10) talked about an increased fear of death—their own or their relatives’.

P3: “*I have a fear of death because there is so much talk on television about people dying of this COVID*”.

P8: “*I feel anxious about what will happen, whether I will get infected or one of my relatives will be a victim of the pandemic*”.

P10: “*I try not to think about it, but I am afraid. I am afraid of dying and of this virus*”.

One patient (P4) noticed that he started to feel better during the pandemic because he had more opportunities to maintain online contact with other people and gained more social support than before the pandemic.

P4: “*Before the pandemic I was isolated from people, but online I found a group of support and friends and I also moved out and started living alone. I had no contact with my father anymore, so it got better for me*”.

A few patients also noticed a positive influence of the pandemic.

P2: “*When I was working from home, I thought it was beneficial for me because I have silence in my apartment and I can just focus on work”.*

P9: “*For example, at home people are more stressed by this disease – afraid that someone may get sick—and this has brought the family together; we spend more time with each other and it is as if we are trying to live well, because nobody knows what might await us in the future*”.

### 3.3. Patients’ Attitudes towards Temporary Limitations and Lockdowns

All the interviewed patients declared that they followed all or most of the temporary pandemic restrictions. However, they presented two different attitudes towards these limitations. Five patients (P4, P5, P8, P9, P10) saw no problems in following the restrictions, or even assessed them as rational and necessary.

P4: “*I think these are wise restrictions since the virus is prevalent. These restrictions are appropriate, and they simply had to be introduced. In my opinion, even too little has been done, because in a pandemic there is no way people should gather in large groups*”.

P9: “*I think they probably reacted well, because in other countries it was much worse and people simply ignored all these restrictions. I think it is necessary. It often happens that people who ignore restrictions suddenly get sick; this opens their eyes and they start to persuade everyone to wear masks and wash their hands. Well, I think you have to be very careful*”.

The other half of the interviewed patients (P1, P2, P3, P6, P7) thought that the limitations were redundant, or they noticed that they had a negative impact on their relations or general mental condition. 

P1: “*These restrictions made me angry. But what was I supposed to do?*”

P2: “*At first they said that masks were not required, but now they say otherwise. This changes frequently and I think it’s wrong. (...) When I don’t meet people, I have a feeling that after some time these relationships will simply break down – that they will find someone else and forget about me*”.

P7: “*I’m a gardener and I was working outside in summer; it was really hard for me to wear a mask all the time, especially when it was really hot. I sometimes didn’t wear one because I couldn’t breathe*”.

### 3.4. Psychiatric Treatment and Psychotherapy during the Pandemic

As the participants were recruited in outpatient treatment services, and all of them had access to psychological and psychiatric help at the time of the interviews. However, they were asked about the first few months of the pandemic and how they experienced the help they received during lockdown. All of the interviewed patients had a chance to talk to their psychiatrists regardless of the pandemic. Contact was mostly maintained by phone. Patients who experienced worsening symptoms (depressive or psychotic) had their pharmacological treatment adjusted. However, access to psychotherapy or therapeutic classes was more limited. Three patients (P1, P4, P10) did not have any access to psychotherapy at all. One patient (P6) used to participate in occupational therapy workshops, but these were temporarily closed due to the pandemic; six patients had psychotherapy limited to phone or Skype calls with temporary breaks. 

P2: “*I am in constant contact with the psychotherapist, but there was a break for some time. My therapist had a sick person at home and therefore I didn’t have therapy. I was tense when this break happened, because something was going on that I wanted to share*”.

P6: “*The workshops were closed and that influenced me. The therapists called to ask how I was feeling, if I needed any help. (...) It did help a bit, but it’s hard, you know*”.

P8: *“I believe that it was a form of therapy that was possible at that time, that is, as much as possible. Nothing else could be offered. Because we were all, so to speak, locked up. So I think I was satisfied*”.

P9: “*Therapy was interrupted due to the coronavirus. And then those phone calls with the psychologist and conversations about the coronavirus certainly helped me survive it all”.*

Table 3 shows the summary of the most common topics that appeared in patients’ reactions to the pandemic.

## 4. Discussion

A general impression arising from the presented study is that the majority of the interviewed patients with a schizophrenia diagnosis coped quite well with stress related to the experience of the COVID-19 pandemic. Only two of the patients (with limited access to psychiatric care) had increased psychotic symptoms due to the pandemic, although these were mild and did not need hospitalization. We could observe that for people living with schizophrenia, the pandemic crisis can be a risk on one hand and a chance on the other, just like for other people. A similar pattern of results was obtained in qualitative research performed in the UK [14]. These contrary aspects of the experience suggest that the pandemic can lead to many risks and challenges, especially in people with severe mental illnesses [33].

When the pandemic was first announced, people all over the world reacted similarly: high levels of anxiety, stress, and depression, or only minimal or mild levels of worry. For example, Wang et al. [12] reported that 53% of Chinese respondents rated the psychological impact of the COVID-19 outbreak as moderate or severe. An Italian study [34] showed that 35% and 73% of respondents experienced symptoms of anxiety or depression, respectively. Other studies also showed that the initial reaction to the pandemic for many people was denial [35] or lack of concern due to misleading information [36]. The results of the presented study seem to show a similar pattern of initial reactions among schizophrenia patients (see Table 3): a total of 50% of the respondents experienced increased levels of anxiety or depression; 50% reacted with disbelief or indifference. In most cases, these reactions changed and adjusted over the course of the pandemic.

A lot of early research assumed that people suffering from schizophrenia could be more vulnerable [20,21]. Meanwhile, the results of this study and the literature review suggest that the responses to the pandemic of people with schizophrenia are comparable to those of healthy people. It could be observed in the present phenomenological analysis of the pandemic experience of people with schizophrenia that the participants thought a lot about being infected. They were worried about relatives and themselves, they felt anxiety and distress on a daily basis, and exhibited increased fear of death. Again, these reactions were not very different from experiences described in other studies. It was reported that, as a result of the pandemic, people often felt pervasive anxiety, frustration, boredom, loneliness, and fear of being infected [37]. Moreover, people experienced loss of social contact and support resulting from isolation [38], and many studies confirmed a higher incidence of various mental health symptoms such as anxiety and depression, distress, sleep disturbance, etc. [4,39,40]. The majority of these studies were qualitative and were mostly centered around the experiences of specific groups, such as health workers [41,42], people hospitalized due to COVID-19 [43,44], children and adolescents [45], and people suffering from various mental illnesses [14,38]. Nonetheless, similar problems were observed in many cases, which had a significant impact on psychosocial functioning [46]. Williams et al. [47] demonstrated that people lost many social interactions as well as structure and routine, which led to psychological consequences in terms of decreasing motivation and self-worth.

Regarding patients’ reactions to the pandemic limitations and restrictions, our study showed a mixture of different reactions: acceptance, willingness to follow all the restrictions in order to feel safer, criticizing incoherent rules, or noticing that some restrictions were difficult to follow. It seems that these reactions are similar to the general population’s and that the pandemic reduced the differences between psychotic patients and the rest of the world [48].

It is characteristic of many patients with schizophrenia to feel socially excluded or isolated [16]. During the pandemic, the difference between them and others suddenly decreased. Most people experienced some level of social isolation [49,50]. Observing how patients coped with the pandemic, it seems that this previous isolation could work as preparation for the pandemic crisis in some cases: “I didn’t care too much because I had a more stay-at-home lifestyle anyway”. We met at least a couple of patients with schizophrenia in our clinical practice who, at the beginning of the pandemic, could act as experts in isolation for their friends or relatives. We also met some patients with chronic delusions who began functioning more normally in the unusual pandemic conditions. For example, one patient, who actually inspired one of the authors to begin this study (unfortunately, he could not participate in the study because he ended his therapy earlier), said about the pandemic that he “always felt that there was something wrong with this world”, and he finally felt normal with his fears and suspiciousness. These clinical observations are consistent with research showing that some patients with schizophrenia presented relatively stable levels of psychotic symptoms and an even better mental condition [51].

On the other hand, social support plays a crucial role in recovery for schizophrenia patients. Social support is gained from casual contacts or various organized communities, but the pandemic changed social behaviors, and isolation suddenly became necessary. This situation can lead to difficulties with establishing spontaneous relations; it can deepen feelings of loneliness or have a negative impact on patients’ mental health [11].

There are still many questions and doubts associated with the standard of psychiatric care during the pandemic [52]; however, our study indicates the importance of continuity of care. Even limited telephone contact was very helpful at times [53]. As one patient said: “I am not left alone, somebody is taking care of me”. Patients noticed the difference between the intensive care offered by occupational therapy workshops or day care centers and the limited online or phone-based support, but they emphasized that it was often crucial to maintaining their mental stability [54]. The majority of patients indicated the importance of being in contact with a psychiatrist, psychologist, or other mental health professional during the pandemic [55]. These results tie in well with previous studies which captured moderate to strong connections between tele-mental health treatment during the pandemic and coping with the pandemic. If respondents felt that their treatment was ongoing, at least by remote sessions, they coped better. In cases of treatment deterioration, when support stopped or patients could not contact professionals, coping was more often impaired [56].

### Limitations and Future Directions

As the presented study is qualitative and focused on the individual experiences of patients suffering from chronic schizophrenia, it contains several limitations. First of all, the nature of the study does not allow for drawing generalized conclusions—but it askes the important questions that seem to be necessary in the times of the pandemic. It can be a first step towards better understanding how patients with schizophrenia experience the COVID-19 pandemic; however, further studies are needed to fully explore the subject. Secondly, qualitative research does not include statistical methods which could provide insight into the correlations between different sociodemographic variables and patients’ reactions to the pandemic. Thus, additional quantitative research with a control group and samples big enough to conduct a statistical analysis might be a future direction worth exploring. Furthermore, the presented study was conducted during the first year of the COVID-19 pandemic. Later, the initial reactions and coping strategies might have changed, so it would be beneficial to conduct a second, comparative study or a long-term study.

## 5. Conclusions

The adopted qualitative methodology and the sample size in the study do not justify generalized conclusions, but it can be observed that most of the interviewed schizophrenia patients’ reactions to the pandemic were quite similar to the reactions of the general population [37,39,40] and people with other mental disorders [14,38]. Patients who experienced more significant changes in their functioning and everyday routine due to the pandemic, such as changes in regular psychiatric care or limited social contact, experienced a greater decline in their mental condition. This is consistent with previous studies [18] which showed that stability in social connections was associated with less severe schizophrenia symptoms and better functioning. The results of this study suggest that their earlier experiences may help people diagnosed with schizophrenia to cope with difficulties such as those encountered during the pandemic. Further work is certainly required to disentangle all the complexities in reactions to the pandemic.

The clinical implications of our study are consistent with the guidelines for psychiatric care during the COVID-19 pandemic proposed in literature [57,58,59]. The organization of health care during the pandemic should take into account that any breaks in contact with patients might have significant impact on their mental state. The psychiatric and psychological help should maintain continuity by any means, including online psychotherapy and telemedicine. Mental health issues related to the pandemic, such as depressive or anxiety symptoms, can occur similarly in the general population and in patients with severe mental illness. Thus, mental health care systems should be prepared to provide services to all in need regardless of preexisting mental disorders.

## Figures and Tables

**Table 1:** Interview questions concerning the pandemic experience.

Question	Expected Aspects of Patient’s Experience	Topic Covered by the Question (Extracted during the Analysis)
How do you perceive the COVID-19 pandemic?	General feelings and thoughts about the pandemic, patient’s main aspects of experience Difficulties and challenges caused by the pandemic	First reaction to information about the pandemic Subjective assessment of the pandemic’s impact on patients’ mental health and functioning
How did you feel when the COVID-19 was declared a pandemic?	Feelings and thoughts that the patient experienced when the pandemic was first declared Subjective experience of the change in perception of the pandemic	First reaction to information about the pandemic
Did you mental state changed since the pandemic started? How?	Experience of the pandemic, subjective assessment of the pandemic’s impact on patient’s mental health Subjective experience of the change in perception of the pandemic	Subjective assessment of the pandemic’s impact on patients’ mental health and functioning
How do you feel about the temporary pandemic restrictions? Did you comply with them?	Patient’s point of view on the pandemic restrictions The perceived impact of the pandemic restrictions on patient’s life	Patients’ attitudes towards temporary limitations and lockdowns
Did the limitations personally affect you? How?	Patient’s point of view on the pandemic restrictions The perceived impact of the pandemic restrictions on patient’s life	Patients’ attitudes towards temporary limitations and lockdowns
How do you feel about your relationships during the pandemic? Did anything change?	The perceived impact of the pandemic on patient’s relationships Necessity to reorganize family life, possible conflicts, the perceived level of social support	Subjective assessment of the pandemic’s impact on patients’ mental health and functioning Patients’ attitudes towards temporary limitations and lockdowns
Did you feel isolated during the pandemic? Did this feeling somehow change because of the pandemic?	The perceived impact of the pandemic on patient’s life and relationships, the perceived level of social support Experience of social isolation before the pandemic, subjective change in this feeling because of the pandemic	Subjective assessment of the pandemic’s impact on patients’ mental health and functioning Patients’ attitudes towards temporary limitations and lockdowns
Can you see any benefits from the pandemic for your personal life?	The perceived changes in patient’s life caused by the pandemic, which might have beneficial impact The perceived level of social support	Subjective assessment of the pandemic’s impact on patients’ mental health and functioning
Did you have access to psychotherapy or other forms of therapy before the pandemic? How did it change when the pandemic had started? How do you feel about the help that you received?	The perceived access to professional help in relation to patient’s needs in this matter Feelings and thoughts about telemedicine and its impact on patient’s mental health	Psychiatric treatment and psychotherapy during the pandemic Patients’ attitudes towards temporary limitations and lockdowns
Did your psychiatrist change your pharmacological treatment during the pandemic? Why?	The perceived access to professional help Subjective assessment of the pandemic’s impact on patient’s mental health	Psychiatric treatment and psychotherapy during the pandemic Subjective assessment of the pandemic’s impact on patients’ mental health and functioning

**Table 2:** Sociodemographic characteristics of the study sample.

Patient	Gender	Age	Work	Living	Diagnosis	History of Illness	Treatment
P1	woman	39	no	with family	Paranoid schizophrenia (F20.0)	more than 10 years	Mental Health Clinic
P2	woman	42	yes	alone	Paranoid schizophrenia (F20.0)	more than 5 years	Mental Health Clinic
P3	man	37	yes	alone	Paranoid schizophrenia (F20.0)	more than 9 years	Mental Health Clinic
P4	man	33	no	alone	Paranoid schizophrenia (F20.0)	more than 14 years	Mental Health Clinic
P5	man	44	yes	with family	Paranoid schizophrenia (F20.0)	more than 13 years	Mental Health Clinic
P6	man	39	no	with family	Paranoid schizophrenia (F20.0)	more than 4 years	Day Ward
P7	man	27	no	with family	Paranoid schizophrenia (F20.0)	more than 9 years	Day Ward
P8	man	38	no	with partner	Paranoid schizophrenia (F20.0)	more than 11 years	Day Ward
P9	woman	36	no	with family	Paranoid schizophrenia (F20.0)	more than 18 years	Day Ward
P10	man	32	no	with family	Paranoid schizophrenia (F20.0)	more than 7 years	Day Ward

**Table 3:** Summary of the most common topics in patients’ reactions to the pandemic.

Initial Reaction to the Pandemic	Subjective Impact of the Pandemic on Mental Health	Attitude towards Lockdown	Access to Psychiatrist and Psychologist	Subjective Assessment of Health Care
indifference	indifference	redundant limitations with negative impact	only psychiatric help	negative impact of breaks in therapy
disbelief	negative impact of isolation on mood	rational and necessary limitations	psychiatric and psychological help with a break	importance of maintaining contact with specialists
depression	negative impact of isolation on personal hygiene	anger	psychiatric and psychological help without a break	positive impact of phone therapy
anxiety	increased fear of death	fully following the restrictions	telemedicine	need for psychotherapy
	gained social support	following only selected restrictions	break in occupational therapy workshops	no need for psychotherapy
	psychotic relapse		changes in pharmacology	
	anxiety			
	feelings of isolation and insecurity			

## Data Availability

The data presented in this study are available on request from the corresponding author. The data are not publicly available due to privacy of the subjects involved in the study.
